# Constructing a novel expression system by specific activation of amylase expression pathway in *Penicillium*

**DOI:** 10.1186/s12934-020-01410-4

**Published:** 2020-07-29

**Authors:** Changyu Pi, Zhe Zhang, Boyu Xiang, Hongwei Tian, Qinzhen Liao, Yu Chen, Liqiu Xia, Yibo Hu, Shengbiao Hu

**Affiliations:** State Key Laboratory of Developmental Biology of Freshwater Fish, Hunan Provincial Key Laboratory for Microbial Molecular Biology, College of Life Science, Hunan Normal University, Changsha, China

**Keywords:** Expression system, Promoter, Transcription factor, Amylase expression, *Penicillium oxalicum*

## Abstract

**Background:**

Filamentous fungi have long been used as hosts for the production of proteins, enzymes and valuable products in various biotechnological applications. However, recombinant proteins are expressed with highly secreted host proteins when stronger promoters are used under inducing conditions. In addition, the efficiency of target protein expression can be limited by the application of constitutive promoters in recently developed filamentous fungal expression systems.

**Results:**

In this study, a novel expression system was constructed by using a *Penicillium oxalium* strain that has powerful protein secretion capability. The secretory background of the host was reduced by knocking out the Amy13A protein and utilizing the starch as a carbon source. The strong promoter *amy15A*(p) was further improved by overexpressing the transcription activator AmyR and deleting of putative repressor CreA. By using the native amylase Amy15A as a reporter, the efficiency of expression from the *amy15A* promoter was dramatically and specifically enhanced after redesigning the regulatory network of amylase expression.

**Conclusions:**

Our researches clearly indicated that the triple-gene recombinant strain Δ13A-OamyR-ΔCreA, with the *amy15A*(p) promoter could be used as a suitable expression system especially for high-level and high-purity protein production.

## Background

Filamentous fungi, as an important expression platform for enzymes and pharmaceutical proteins, have the advantages of low requirements for raw materials, proper glycosylation and post-translational modification of eukaryotic proteins compared with *Escherichia. coli* and *Saccharomyces cerevisiae*. Moreover, filamentous fungi have a high-density growth adaptability and excellent protein expression; therefore, they are widely used in industry. The secretory potential of filamentous fungi such as *Aspergillus* sp. and *Trichoderma* sp. was reported to be approximately ten times higher than that of *S. cerevisiae*. For example, the hypersecreting mutant *Trichoderma reesei* RUT-C30 could express more than 100 g/L total protein [1, 2]. The production of a single enzyme secreted by *Aspergillus niger* also reached the gram per liter level [3].

Proteins of interest are commonly expressed by hypersecreting mutants with strong host promoters. For example, a large number of recombinant proteins, such as human erythropoietin, antibody and hydrolases have been successfully expressed by using *cbhI* promoter in *T. reesei* and *glaA* promoter in *A. nige*r, respectively [4–7]. However, the secretion of a large number of extracellular native proteins by the host could easily affect the purification and identification of target proteins. Meanwhile, the relatively low proportion of fermentation proteins increases the costs of the final products. Recently, low background host strains have been achieved by inhibiting extracellular protein secretion through strategies such as knocking out the cellulases transcriptional activator Xyr1 in *T. reesei* or utilizing glucose as non-inducer carbon source for cultivation [8, 9]. Similar genetic modifications were performed in *A. niger* to repress the expression of extracellular amylase [10]. Then, the target protein could be expressed at a relatively high purity by using constitutive promoters. Generally, the key factor for the high yield of proteins or natural products is mostly the selected strong promoters. Constitutive promoters, such as *gpdA*(p) or *tef1*(p), have been reported and widely used for protein expression in filamentous fungi [9, 11]. However, further improvements for strong constitutive promoters have been restricted since their regulatory network in the host remains unknown. Therefore, an expression system that including chassis cells with high expression efficiency, low extracellular background, strong promoters and further improvement potential, could break the bottleneck for the broad application of filamentous fungi for protein expression.

*Penicillium oxalicum* has been studied and applied for commercial cellulases production in China for more than 20 years [12]. In particular, an engineering strain RE-10, constructed from the wild-type (WT) strain 114-2 with three gene modifications, resulted in nearly equal cellulases production and higher extracellular protein secretion compared to the industrial hypersecreting mutant JU-A10-T [13]. Their study suggested that WT 114-2 has the potential to become a high efficient secretory host cell by genetic engineering. Secretome analysis revealed that amylase Amy15A and cellulase Cel7A-2(CBHI) were the two most abundant extracellular proteins in the WT strain after induction, suggesting that the Amy15A expression pathway had the same transcription and secretory efficiency as the CBHI expression pathway [14]. Previous studies showed that Amy15A and Amy13A were the two major proteins expressed on glucose since cellulases were not induced. Hu et al. found that starch could induce the expression of amylase, which also considered to be activated by transcriptional activator AmyR in *P. oxalicum* [15]. The carbon catabolite repressor CreA was proven to regulate various biological processes, mainly cellulase expression, in many filamentous fungi [16, 17]. Although the role of CreA in regulating amylolytic genes has not yet been systematically studied, some experiments have shown that deletion of CreA leads to enhanced amylase activity in *P. oxalicum* [16].

In this study, we specifically activated the Amy15A pathway by applying strategies of carbon source optimization and genetic modification of regulators through gene overexpression and deletion in *P. oxalicum*. Finally, a low background expression host strain and high efficiency promoter were obtained for protein expression.

## Results

### Deletion of Amy13A reduced the extracellular protein background and made the native amylase Amy15A a reporter

**Fig. 1:**
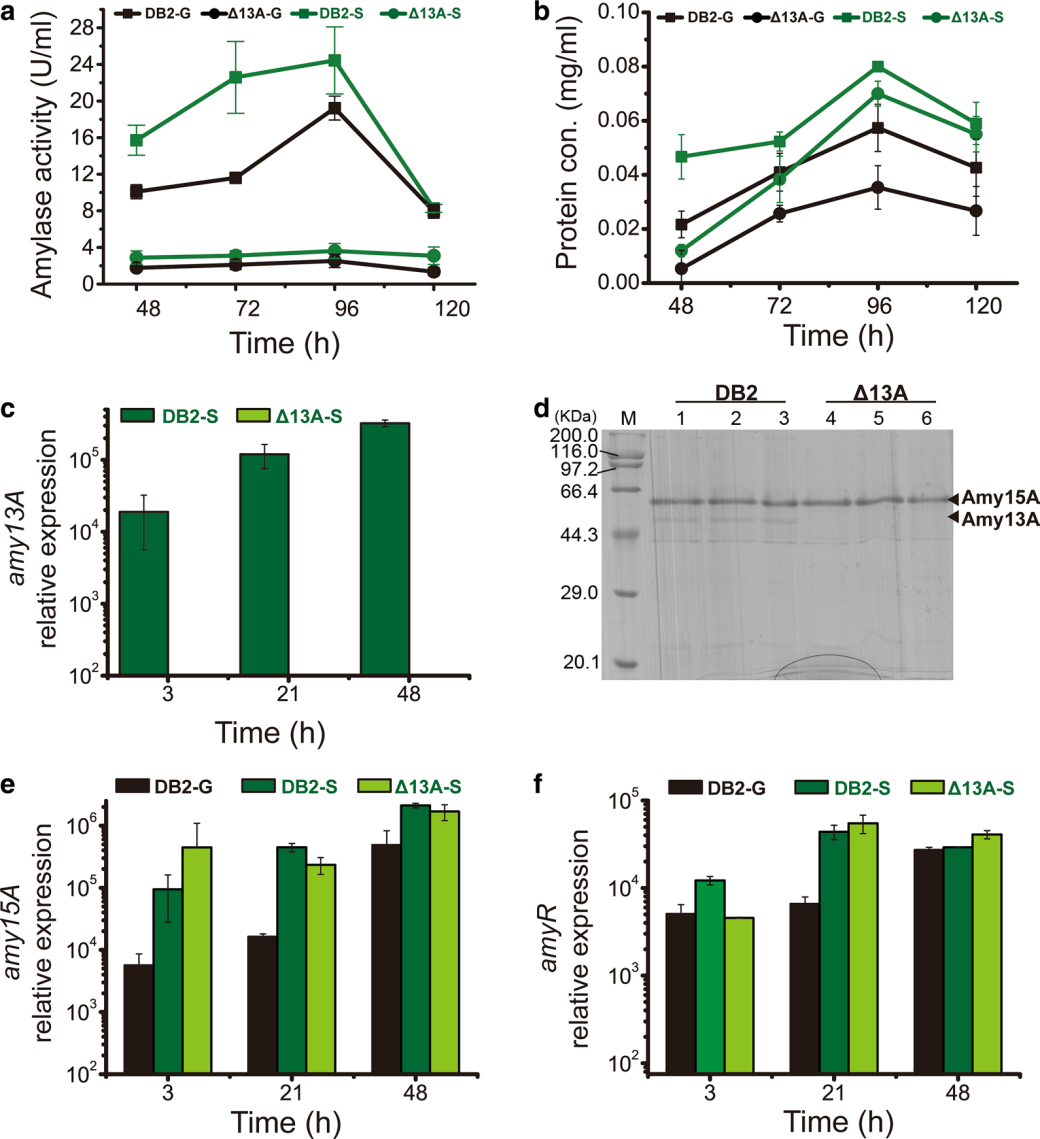
Amylase activity, protein concentration, SDS-PAGE and transcription level analyses of strains DB2 and Δ13A on different carbon sources. **a** Amylase activity of strains DB2 and Δ13A on glucose (-G, labeled in black) and starch (-S, labeled in green). **b** Protein concentration of strains DB2 and Δ13A on glucose (-G, labeled in black) and starch (-S, labeled in green). **c** Expression levels of the *amy13A* in strains DB2 and Δ13A on starch. **d** SDS-PAGE analysis of extracellular protein of DB2 and Δ13A on starch, every three lanes represent three independent cultivations for each strain. Expression levels of the *amy15A* (**e**) and *amyR* (**f**) genes in strains DB2 and Δ13A on glucose and starch

The genome annotation and analysis identified five amylases, Amy15A (PDE_09417), Amy15B **(**PDE_05527), Amy13A (PDE_01201), Amy13B (PDE_01021) and Agl31A (PDE_03966) in WT 114-2 [18]. Secretomes were analyzed by LC–MS/MS, and the results showed that Amy15A and Amy13A, which occupied 28.85% and 10.95% of the total protein, respectively, were two major amylases in the WT strain when cultured on cellulose [14]. Although the secretion of Amy13A was much lower than that of Amy15A, deletion of this gene resulted in dramatic decrease in amylase activity. However, it has also been reported knocking out Amy13A or both Amy13A and Amy15A does not affect vegetative growth [15]. Considering the subsequent strategies of genetic engineering on amylase expression activation, Amy13A, a redundant extracellular protein that would be enhanced after activation, was knocked out to obtain a cleaner background. Therefore, Amy15A, the major extracellular protein could be used as a reporter to evaluate the efficiency of its promoter *amy15A*(p). As shown in Fig. 1a, the amylase activity of the Δ13A strain was significantly decreased regardless of whether the strains were cultured on glucose or starch. On the other hand, the extracellular protein concentration of Δ13A was reduced under both carbon sources compared to that of the parent strain DB2 (Fig. 1b). Besides, the application of targeted metabolic engineering to improve recombinant protein production has been carried out in yeast systems and *Aspergillus* [19, 20]. Therefore, the deletion of Amy13A to prevent bypass metabolism may result in enhanced production of Amy15A protein. However, in this experiment, the abundance of Amy15A in the Amy13A-deletion strain Δ13A did not increase according to qRT-PCR detection and SDS-PAGE analysis (Fig. 1c, d). Compared with glucose as the sole carbon source, enhanced amylase activity and protein concentration were observed in the presence of starch, regardless of whether Amy13A was present (Fig. 1a, b).

The effect of the two carbon sources (glucose and starch) and Amy13A deficiency on the expression of Amy15A was investigated. qRT-PCR was performed to detect the transcript levels of *amy15A* and *amyR* in the DB2 and Δ13A strains after 3 h, 21 h and 48 h of cultivation on glucose or starch medium. The transcript levels of *amy15A* were increased by 16.8-, 27.5- and 4.3-fold on starch compared with glucose at the three detection points, respectively (Fig. 1e). Meanwhile, the transcript levels of *amyR* were increased by 2.4-, 6.6- and 1.1-fold, respectively (Fig. 1f). The results revealed that the starch could better activate the amylase expression pathway than that of glucose. However, compared with the expression in parent strain DB2, no significant difference in transcription of *amy15A* or *amyR* was observed after deleting Amy13A when cultured on starch (Fig. 1e, f).

### Further enhancement of *amy15A*(p) transcription by overexpression of AmyR with the *amy13A* promoter

**Fig. 2:**
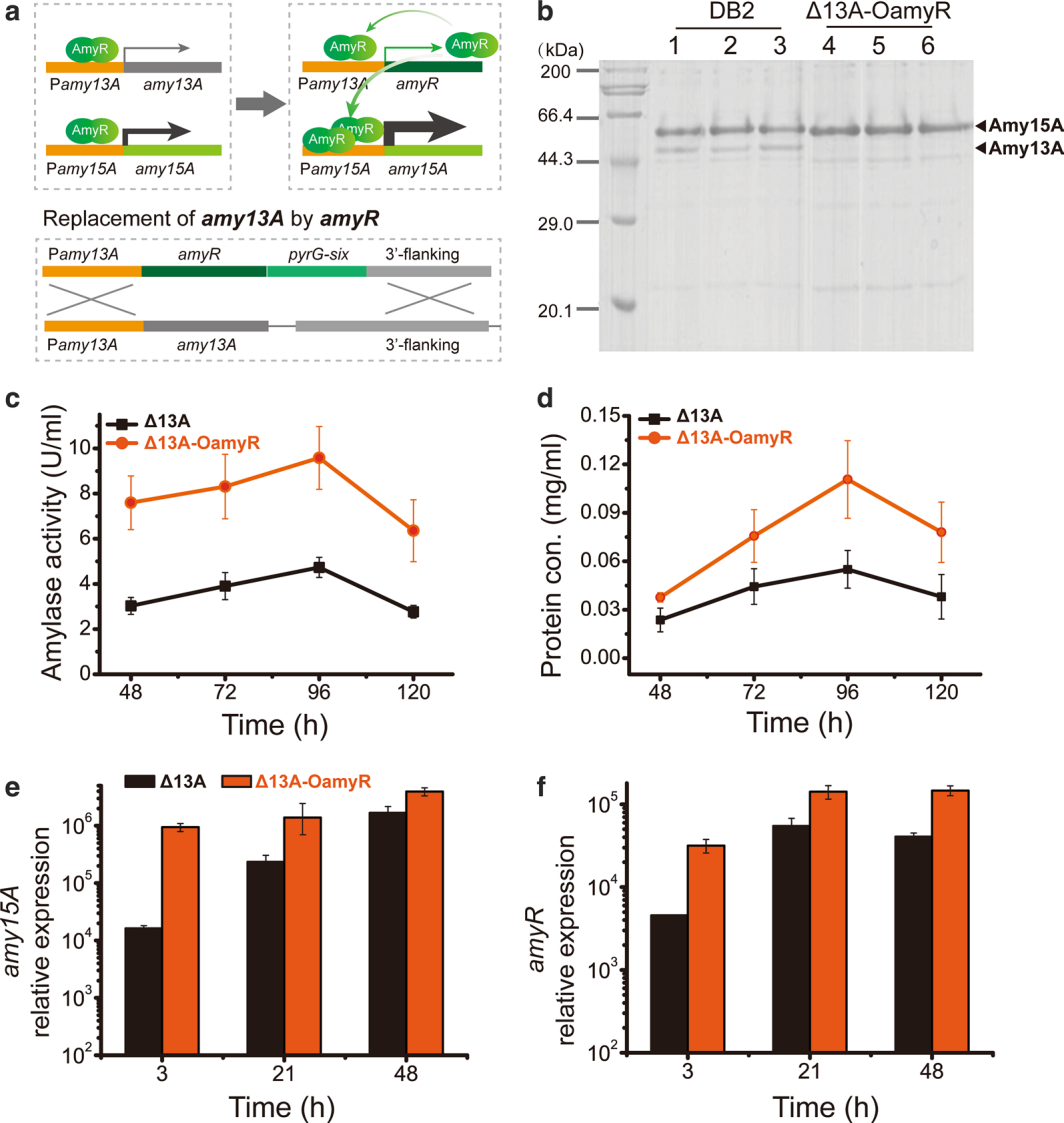
SDS-PAGE, amylase activity, protein concentration and transcription level analyses of strains DB2, Δ13A and Δ13A-OamyR. **a** Strategy and schematic diagram of the construction of Δ13A-OamyR by using DB2 as the parent strain. **b** SDS-PAGE analysis of extracellular protein of DB2 and Δ13A-OamyR on starch, every three lanes represent three independent cultivations for each strain. Amylase activity (**c**) and protein concentration (**d**) of strains Δ13A and Δ13A-OamyR. Expression levels of the *amy15A* (**e**) and *amyR* (**f**) genes in strains Δ13A and Δ13A-OamyR on starch

The transcription factor AmyR was identified as an activator for the expression of amylase. The lack of AmyR significantly decreased the expression of amylase genes involved in starch degradation [16]. Given that the two amylases, Amy13A and Amy15A, might both be positively regulated by AmyR, the coding region of *amyR* was applied to replace the *amy13A* coding region for overexpressing AmyR by *amy13A* promoter. This strategy might promote the expression of a” cascade amplification” by AmyR (Fig. 2a). As shown in Fig. 2b, SDS-PAGE analysis confirmed that the Amy13A bands disappeared and that the Amy15A bands were obviously enhanced in the Δ13A-OamyR overexpression-knockout mutant compared to that in the DB2 strain.

To clarify the effect of AmyR overexpression on *amy15A* expression, amylase activity and protein concentration in strain Δ13A-OamyR were determined after induction with soluble starch, and Δ13A was chosen as the reference strain. The results showed that amylase activity in Δ13A-OamyR increased by 202%–251% compared to that in Δ13A (Fig. 2c), and the concentration of extracellular protein was upregulated by 159%-205% during the entire induction period (Fig. 2d). Moreover, the expression patterns of *amy15A* and *amyR* were analyzed by qRT-PCR in Δ13A and Δ13A-OamyR. The two genes were upregulated remarkably in Δ13A-OamyR, the expression of *amy15A* in Δ13A-OamyR were 57.9-, 5.9-, and 2.3-fold higher than that in Δ13A at 3 h, 21 h and 48 h, respectively (Fig. 2e). Meanwhile, the expression of *amyR* in Δ13A-OamyR were 6.9-, 2.6-, and 3.6-fold higher than that in Δ13A at 3 h, 21 h and 48 h, respectively (Fig. 2f).

### Deletion of CreA dramatically increased *amy15A*(p) transcription levels

**Fig. 3:**
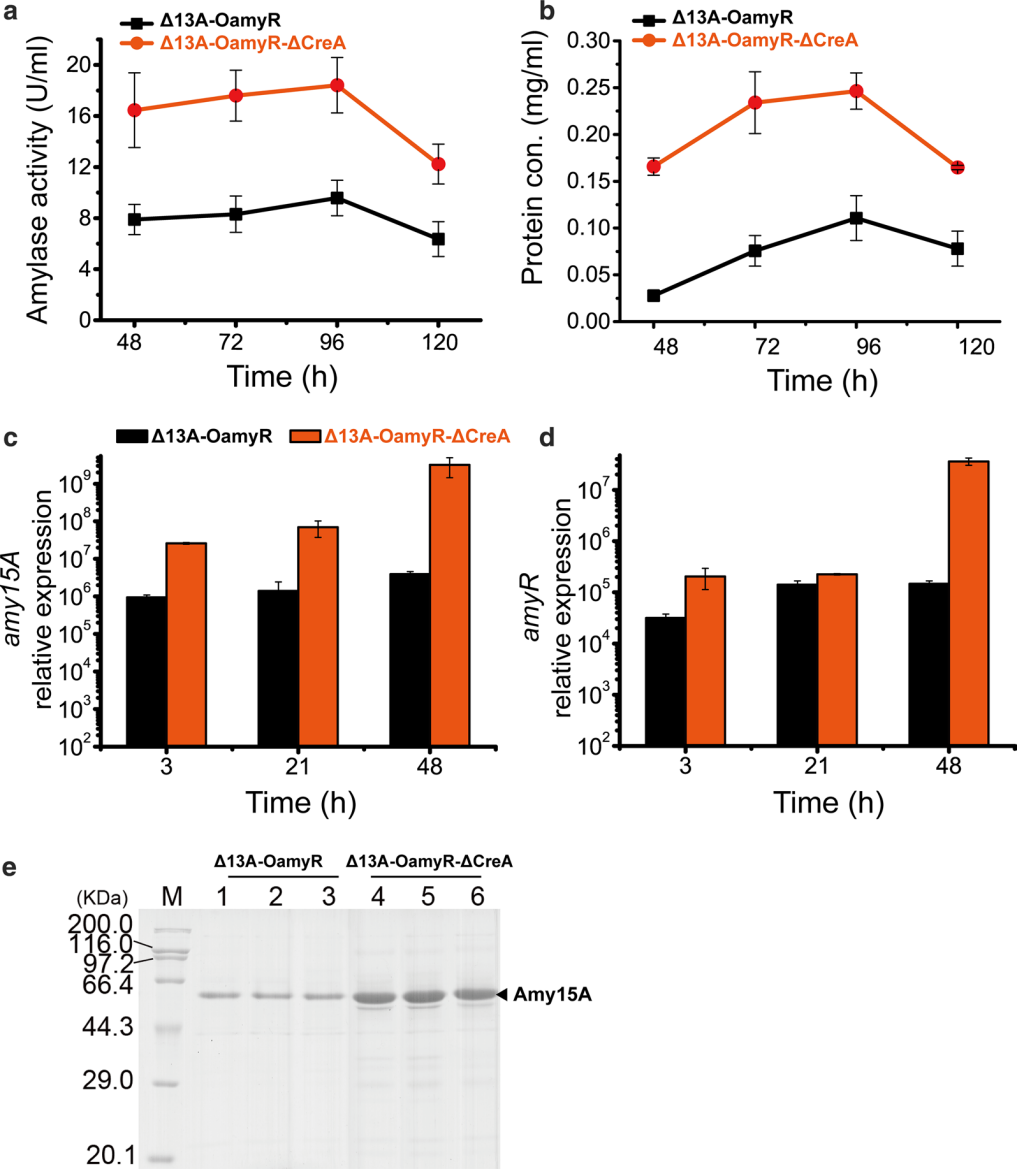
Amylase activity, protein concentration, transcription level and SDS-PAGE analyses of strains Δ13A-OamyR and Δ13A-OamyR-ΔCreA. Amylase activity **(a)** and protein concentration (**b**) of strains Δ13A-OamyR and Δ13A-OamyR-ΔCreA. Expression levels of the *amy15A* (**c**), *amyR* (**d**) gene in strains Δ13A-OamyR and Δ13A-OamyR-ΔCreA on starch. (**e**) SDS-PAGE analysis of extracellular protein of Δ13A-OamyR and Δ13A-OamyR-ΔCreA on starch, every three lanes represent three independent cultivations for each strain

CreA is a wide-domain master regulator of carbon metabolism and has been identified in filamentous fungi. The function of CreA homologs in repressing cellulolytic and xylanolytic gene expression is conserved among cellulolytic fungi. Deletion of CreA resulted in increased amylase, as reported in previous *P. oxalicum* studies [16]. However, there was no direct experimental evidence about whether the lack of CreA could enhance the transcription of *amy15A*. Therefore, in order to investigate the regulatory roles of CreA in the expression of *amy15A* gene, a CreA knockout cassette was constructed by double-joint PCR and introduced into the parent strain Δ13A-OamyR after eliminating its *pyrG* gene by a β-rec/six self-excising marker recycling system. The transformant Δ13A-OamyR-ΔCreA was obtained and showed a 192%–212% increase in amylase activity (Fig. 3a), and its concentration of extracellular protein which mainly included Amy15A, was significantly enhanced by 211%–599% compared with strain Δ13A-OamyR (Fig. 3b). Moreover, qRT-PCR results showed that the transcript levels of *amy15A* in Δ13A-OamyR-ΔCreA were increased by 27.6-, 50.0-, 814.2-fold at 3 h, 21 h and 48 h compared with those in the parent strain, respectively (Fig. 3c). Meanwhile, the transcript levels of *amyR* in Δ13A-OamyR-ΔCreA were increased by 6.4-, 1.6-, 246.1-fold at those three detection points compared with those in the parent strain, respectively (Fig. 3d). Surprisingly, *amy15A* expression was almost specifically enhanced except a protein band with a MW of approximately 60 kDa was slightly secreted according to the SDS-PAGE analysis when cultured on starch medium (Fig. 3e).

### Influence on mycelium morphology of the mutant strains

**Fig. 4:**
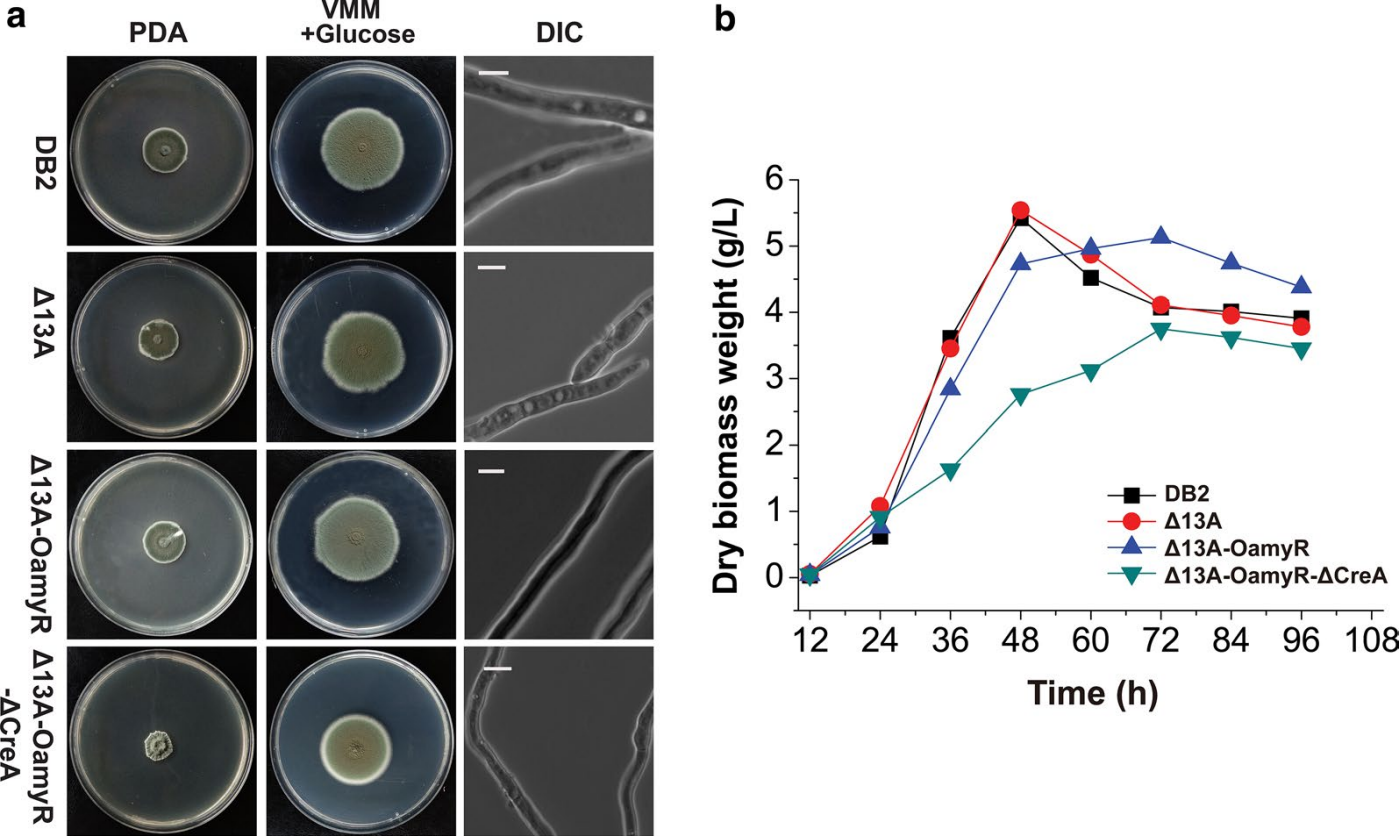
Colony morphology and biomass of DB2, Δ13A, Δ13A-OamyR and Δ13A-OamyR-ΔCreA. **a** The plates were incubated at 30 °C for 6 days. Mycelia grown in GMM for 12 h were subjected to differential interference contrast (DIC) microscopic analysis. Bar = 400 μm. **b** Biomass of strains DB2, Δ13A, Δ13A-OamyR and Δ13A-OamyR-ΔCreA grown on starch medium

Equal amounts of fresh spores collected from *P. oxalicum* DB2, Δ13A, Δ13A-OamyR and Δ13A-OamyR-ΔCreA were inoculated on solid-medium plates in the presence of glucose as the sole carbon source and potato dextrose agar (PDA), and cultured at 30 °C for 6 days. The colonies of Δ13A-OamyR-ΔCreA were smaller in size, while the colonies of strains Δ13A and Δ13A-OamyR showed no obvious change when compared with the parent strain DB2 on PDA and glucose plates. The presence of smaller and thicker Δ13A-OamyR-ΔCreA colonies on PDA indicates that the deletion of CreA may lead to a reduction in hypha expansion ability. In liquid medium with glucose as the sole carbon source, the hyphae of Δ13A-OamyR-ΔCreA appeared thinner than those of the parent strain, while no obvious morphological change in hyphae was observed for the Δ13A and Δ13A-OamyR strains (Fig. 4a).

Additionally, we measured the mycelial biomass of the four *P. oxalicum* strains grown in liquid starch media. The mycelial weight of Δ13A grown in starch medium was similar to that of DB2. Higher maintenance of mycelial biomass was observed in Δ13A-OamyR at the later phase of cultivation. The conidia of the Δ13A-OamyR-ΔCreA strain germinated and grew much slower than those of the Δ13A-OamyR strain in liquid starch culture (Fig. 4b).

### Transcriptome analysis of the response of *P. oxcalium* strains to overexpression of AmyR and deletion of CreA

**Fig. 5:**
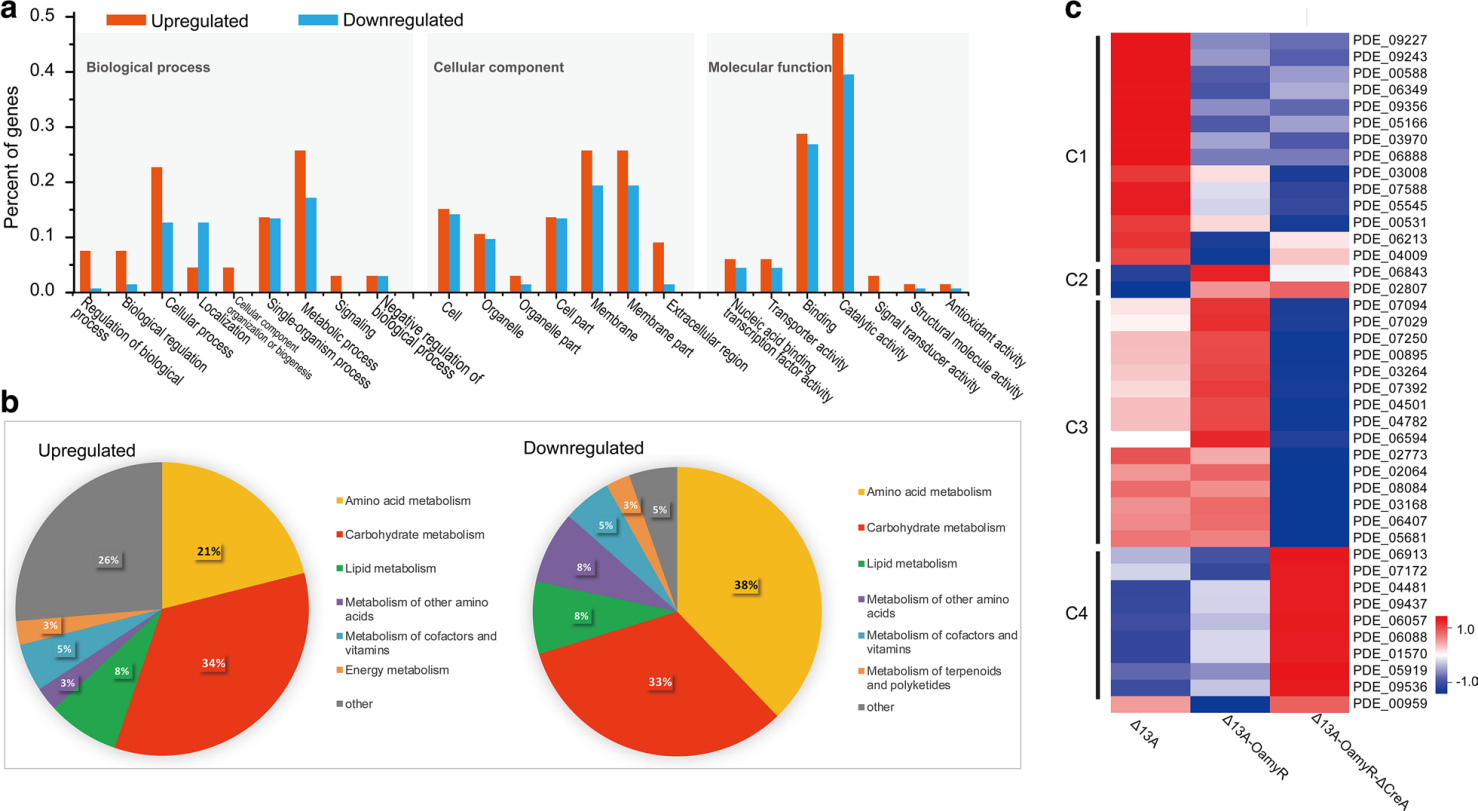
Comparative analysis of the transcriptomes of Δ13A, Δ13A-OamyR and Δ13A-OamyR-ΔCreA cultivated in starch medium. **a** GO enrichment analysis of continuously upregulated and downregulated genes in strains in Δ13A, Δ13A-OamyR and Δ13A-OamyR-ΔCreA strains. Red bars and blue bars indicate the function enrichment analysis of upregulated and downregulated gene sets, respectively. **b** Kyoto encyclopedia of genes and genomes (KEGG) annotation of continuously upregulated and downregulated genes in strains in Δ13A, Δ13A-OamyR and Δ13A-OamyR-ΔCreA strains. **c** Heatmap showing the transcription levels of DEGs encoding putative transcription factors in Δ13A, Δ13A-OamyR and Δ13A-OamyR-ΔCreA strains

To gain insight into the molecular mechanism that underlies the regulation of *amy15A* expression by AmyR-CreA on starch, we evaluated the global changes in the Δ13A, Δ13A-OamyR, and Δ13A-OamyR-ΔCreA mutants by RNA-Seq with three biological replicates. Every sample produced approximately 50 million clean reads, with a < 0.025% error rate and > 92.5% mapped into the genome of strain 114-2. The high consistency of samples among three biological replicates made the transcriptome data more reliable (Additional file 1: Table S1). By using an FDR ≤ 0.01 and fold change ≥ 2 as the threshold, we determined that 680 genes were upregulated and 1042 genes were downregulated in response to the Δ13A-OamyR strain compared with the Δ13A strain (Additional files 2 and 3: Table S2 and S3). In addition, we also found that 762 genes were upregulated and 935 genes were downregulated in response to the Δ13A-OamyR-ΔCreA strain compared with the Δ13A-OamyR strain (Additional files 4 and 5: Table S4 and S5). Among the above DEGs, we were interested in the genes that were positively and negatively related to the improvement in *amy15A* transcription. Consequently, we analyzed the genes that were continuously upregulated or downregulated successively in strains Δ13A, Δ13A-OamyR and Δ13A-OamyR-ΔCreA. 66 genes and 134 genes were upregulated and downregulated respectively, in response to the overexpression of AmyR and subsequent deletion of CreA (Additional files 6 and 7: Table S6 and S7). GO analysis revealed that both upregulated and downregulated genes were enriched in the molecular function of catalytic activity (GO:0003824) and binding (GO:0005488) (Fig. 5a), demonstrating that AmyR and CreA mainly regulated the expression of glycoside hydrolases. Furthermore, KEGG annotation indicated that these DEGs were primarily involved in metabolism, specifically amino acid metabolism, carbohydrate metabolism and lipid metabolism (Fig. 5b).

Generally, glycoside hydrolases expression is regulated by upstream transcription factor(s) in filamentous fungi [16, 21, 22]. Comparative analysis of significantly changed genes in all three strains revealed 40 DEGs encoding putative transcription factors (TFs) (Additional file 8: Table S8), most of which contained zinc-related structures (Zn_clus, GATA, Zn2Cys6, bZIP, etc.). As shown in Fig. 5c, Cluster 1(C1) consisted of 14 TF genes displaying low expression while C2 consisted of 2 TF genes displaying high expression in strain Δ13A-OamyR or Δ13A-OamyR-ΔCreA. Among these 40 DEGs, 15 TF genes (C3) showing low expression and 9 TF genes (C4) showing high expression only in strain Δ13A-OamyR-ΔCreA. These data clearly indicated the transcription of the putative TFs were regulated in different modes. In particular, of these genes, *PDE_04481* (Zn_clus), *PDE_09437*, *PDE_06057* (Zn_clus), *PDE_06088* (Zn_clus) and *PDE_01570* (Zn_clus) were found to be continuously upregulated while *PDE_03008* (Myb_DNA-binding), *PDE_07588* (TF_Zn_Ribbon), *PDE_05545* and *PDE_00531* (Zn_clus) were found to be continuously downregulated in Δ13A-OamyR and Δ13A-OamyR-ΔCreA, respectively (Fig. 5c). Additionally, since the protein (Amy15A) secretion has been enhanced in strain Δ13A-OamyR and Δ13A-OamyR-ΔCreA, we globally monitored the transcriptome of the three strains to further investigate the protein secretory pathway. By using the secretory model of *S. cerevisiae* as a scaffold, 60 genes were mapped to its secretory components through homology search [23]. The transcription of the 60 homologous genes which involved in translocation, ERAD (ER-associated degradation), protein folding, glycosylation, Golgi processing, protein localization (COPI, COPII, etc.) have been analyzed. As a result, with the enhancement of protein secretion, no significant changes have been found in these genes expression except the gene *PDE_07822* (involved in ERAD process) downregulated in Δ13A-OamyR-ΔCreA and the gene *PDE_01584* (involved in glycosylation process) upregulated in Δ13A-OamyR and Δ13A-OamyR-ΔCreA, respectively (Additional file 9: Table S9).

**Fig. 6:**
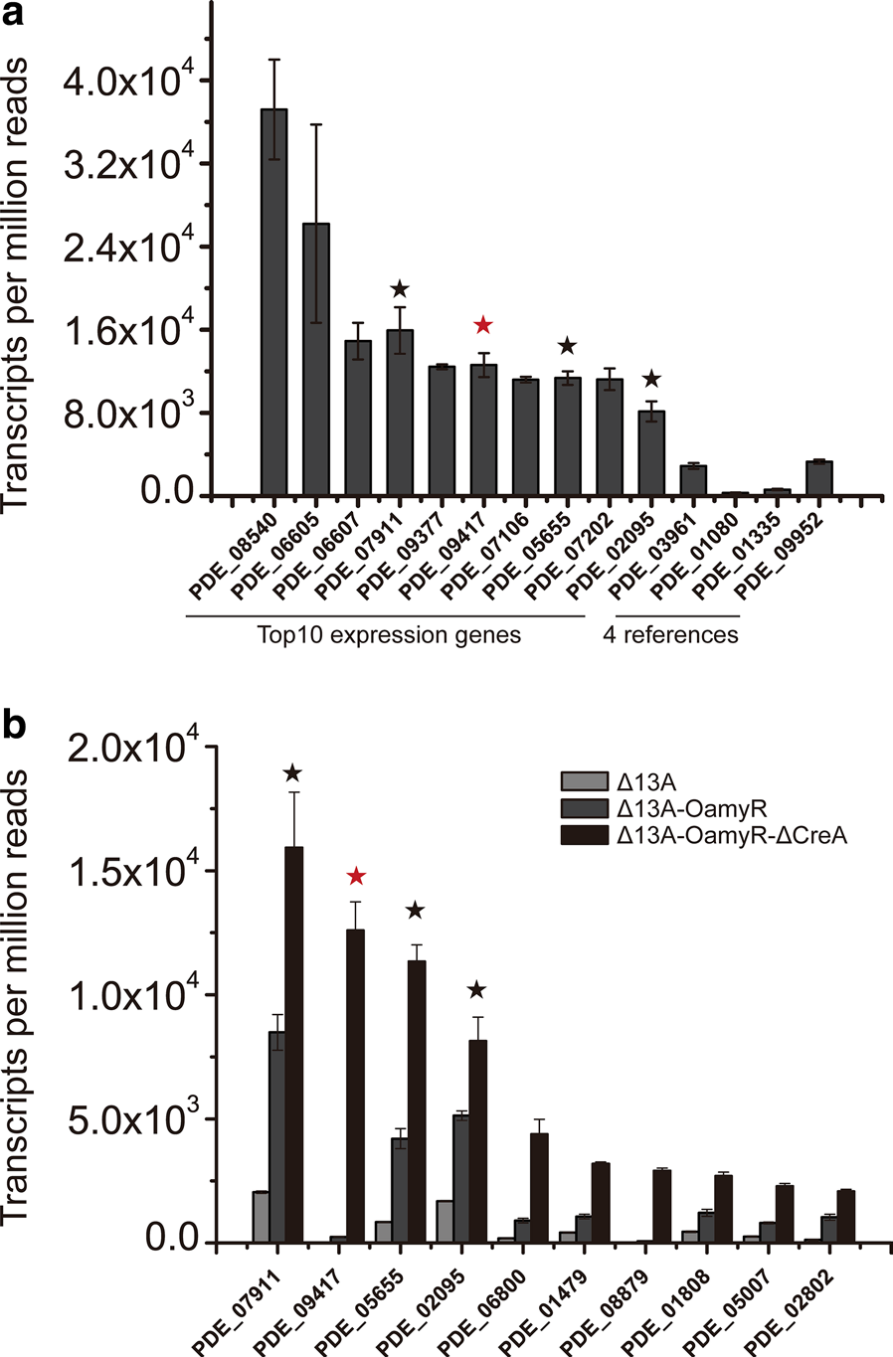
Genome-wide transcriptome analysis for Δ13A, Δ13A-OamyR and Δ13A-OamyR-ΔCreA cultivated in starch medium. **a** Expression levels of the top 10 genes and 4 identified stronger constitutively expressed genes in strain Δ13A-OamyR-ΔCreA. The copy number of unambiguous transcripts for each gene was normalized to TPM. **b** Expression levels of the top 10 genes, which were continuously upregulated in strains in Δ13A, Δ13A-OamyR and Δ13A-OamyR-ΔCreA strains. The stars indicate the same genes involved in both graph **a** and **b**, The red stars indicate the *amy15A* gene (PDE_09417)

Promoters are crucial regulatory elements for controlling protein production and can largely affect gene expression at the transcriptional level. Although the promoter *amy15A*(p) was identified as the primary choice for protein expression, the more strong promoters appear to result in more options for high protein expression yields. Therefore, we determined the 10 most strongly expressed genes with the top 10 TPM values in Δ13A-OamyR-ΔCreA (Additional file 10: Table S10). As expected, *amy15A* (PDE_09417) was one of these 10 genes, and the transcript levels from *amy15A*(p) was far beyond those of the former identified constitutive promoters including *ubiD*(p) (PDE_03961), *pgmC*(p) (PDE_01080), *aciA*(p) (PDE_01335) and *gpdA*(p) (PDE_09952) (Fig. 6a) in *P. oxalicum* [15]. The combination of qRT-PCR results with the TPM data indicated that the transcription efficiency of *amy15A*(p) was significantly enhanced. In addition, the expression of three other genes, PDE_07911, PDE_05655 and PDE_02905, were found to be continuously upregulated in Δ13A, Δ13A-OamyR and Δ13A-OamyR-ΔCreA, demonstrating that those genes may be regulated in the same pattern as *amy15A* and could be used as candidate promoters (Fig. 6b).

## Discussion

Nowadays, the development of filamentous fungus expression systems has become a research hotspot in the field of protein expression throughout the world. Recombinant proteins expressed in *Aspergillus* sp. and *Trichoderma* sp. are commonly secreted with native proteins, such as glucoamylases in *Aspergillus* sp. [11]. Genetic modifications by knocking out proteases and the majority of extracellular proteins, such as cellulase Cel7A/Cel6A, or multiple-round mutagenesis, were performed in the host strain and attempted to gain a cleaner background for target proteins expression [24]. Obviously, it is very cumbersome to obtain low background hosts by mutagenesis which lead to further disordered regulation network and more sensitive chromosome repair system in hypersecreting mutants. Genomic modifications including multiple extracellular protein genes deletion should base on sufficient screening marker genes. Although the CRISPR/Cas9 system was recently developed in filamentous fungi [25], the construction process was found to be more complicated when compared with the classic methods of homologous recombination. Similar to *Trichoderma* sp. and *Aspergillus* sp., *P. oxalicum* strains possess extraordinary secretion capability of fungal cellulases under inducing substrates [13, 26]. In our study, cellulases expression were repressed by using the starch as the suitable carbon source which was beneficial for the transcription from *amy15A*(p) simultaneously. The deletion of Amy13A led to further reduced extracellular protein background in *P. oxalicum* host cell that few proteins secreted on starch. Furthermore, we suggested that the application of the native amylase Amy15A as a reporter for stronger promoter efficiency might be more appropriate than the application of a fluorescent protein, which may lead to fluorescence saturation after excessive accumulation. Meanwhile, the extracellular amylase reporter is advantageous because it could detect the secretory machinery of the host cell.

The homologs of regulator Xyr1 and AmyR were identified as essential transcription activators in cellulases and amylases expression in *T. reesei* and *A. niger*, respectively [27, 28]. Therefore, the strategy that deletion of transcription activators (Xyr1/AmyR) to depress the downstream extracellular proteins expression was adopted to obtain a host with lower background for protein expression in recent years. Transcription factors, ClrB, XlnR, AmyR and CreA were previously identified as the major regulators of glycoside hydrolases in *P. oxalicum.* Particularly, AmyR was proven to possibly control the balance between cellulolytic and amylolytic gene expression [16]. Therefore, overexpression of AmyR not only upregulated the transcription of Amy15A but also depressed the expression of cellulases which is considered an extracellular redundant protein in this study. Thus, the strength of *amy15A*(p) was specifically enhanced by using the AmyR-*amy15A*(p) regulatory/induction pathway. In contrast, the efficiency and further improvement of the promoter *cbhI*(p) or *glaA*(p) were restricted in the genetically modified hosts *T. reesei* and *A.niger*, in which Xyrl or AmyR were deleted to reduce the secretion of cellulases or glucoamylases [9, 10]. The transcription levels of *amy15A* and *amyR* have not been enhanced as higher as we expected when using promoter *amy13A*(p) for AmyR overexpression (Fig. 2). The results indicate the genetic modification strategy shown in Fig. 2a may not work or the AmyR-*amy15A*(p) regulatory/induction pathway is still under partial inhibition.

CreA homologs generally play an important role by linking CCR to developmental programs, including the conidia formation and hyphal morphology in *P. oxalicum* [16]. The significantly upregulated expression of *amy15A* and *amyR* after knocking out CreA demonstrated that the two genes expression were repressed by CreA. Therefore, the dramatic increase in the transcription levels of *amy15A* benefited from not only the absence of the repressor CreA but also the increase in the activator AmyR. In view of this conclusion, it is desirable to further improve the efficiency of *amy15A*(p) by overexpressing AmyR or other potential activitors when inhibition effect mediated by CreA is relieved [22]. In addition, the negative regulation of CreA on hyphal growth and enhanced secretion of Amy15A in liquid indicate that Δ13A-OamyR-ΔCreA has higher expression efficiency per unit biomass, which would benefit protein productivity in high-density fermentation.

*P. oxalicum* produces large amounts of glycoside hydrolases, most of their production is tightly controlled by complex regulatory networks [16, 29]. Besides AmyR and CreA, other novel regulators of amylolytic enzymes were screened and identified in *P. oxalicum* through transcriptional profiling and genetic analysis [22]. The differentially expressed TFs in response to the AmyR overexpression and CreA deletion indicated these TFs might directly or indirectly regulate the efficiency of *amy15A*(p) in this study (Fig. 5c). The results clearly demonstrated that those TFs genes would become the targets of genetic engineering to further improve the *amy15A* promoter in future studies. Although the extracellular protein (Amy15A) secretion was gradually improved, the transcription of secretory component genes was found to be nearly no significant changes among the engineered strains. The fact indicated that the current high-level Amy15A expression had not yet triggered secretory stress response at the transcription level mainly due to efficient secretion capacity of *P. oxalicum.*

At present, the efficient expression of recombinant protein depends largely on promoter selection strategy. High protein production is usually achieved by construction of expression cassettes with stronger or multiple promoters. Moreover, the strength of promoters can be further improved by genetic modification, which includes deleting of repressor binding sequences and adding of activator binding sequences [30]. In addition, a novel and broad-spectrum synthetic promoter was established based on the knowledge of transcription factors and their binding sites [31]. In our work, the strength of *amy15A*(p) was dramatically enhanced by genetic modification of the repressor and activator in the host strain. Since genetic manipulation in the present study was performed at the “host” level, it provided more space for further improving the efficiency of expression at the “promoter” level that are described above; for example, multiple copy integration or artificial synthetic promoter construction. Moreover, the significantly differentially expressed genes detected by transcriptomics analysis of the three recombinant strains might be used to provide new knowledge about the *amy15A*(p) regulation mechanism, and such knowledge would provide more strategies to further improve host strain in the future.

## Conclusions

**Fig. 7:**
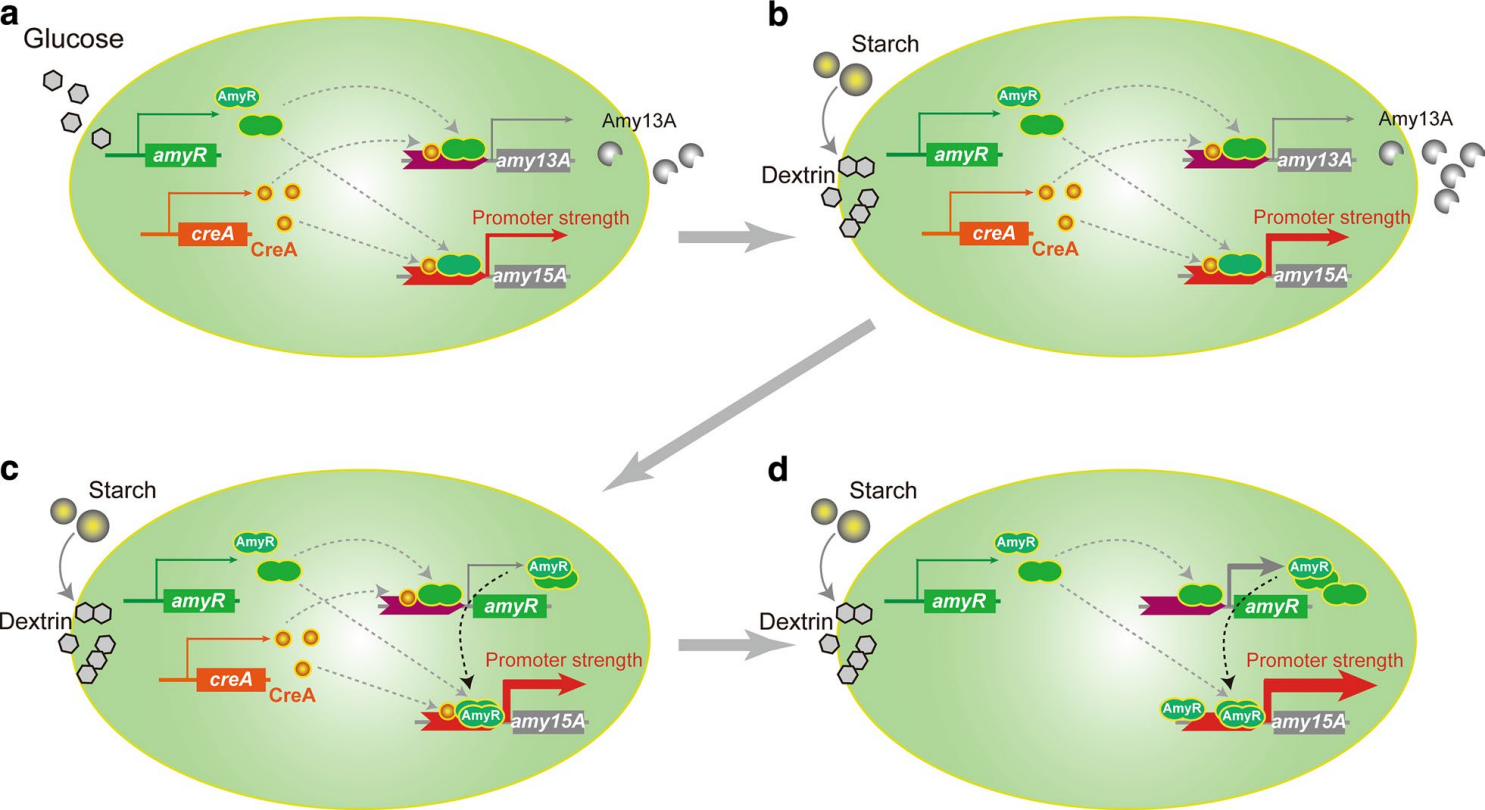
Scheme of a systematic strategy for expression system construction of *P. oxalicum*. The secretory background of host was reduced by knocking out the Amy13A protein and selecting the starch as carbon source (**a**) (**b**). The promoter P*amy15A* strength were further improved by overexpression the transcription activator AmyR (**c**) and deletion of putative depressor CreA (**d**)

In this study, we optimized the carbon source for cultivation and genetically modified three key genes to redesign the regulatory network for the expression of *amy15A* amylase pathway (Fig. 7). The genetically engineered strain exhibited approximately 156 ~ 6580-fold higher *amy15A*(p) transcription levels than that of initial state (Additional file 11: Figure S1). Analysis of extracellular protein revealed that the reporter Amy15A was nearly specifically enhanced in the Δ13A-OamyR-ΔCreA strain. Therefore, an expression system comprising a host strain with a low protein secretion background and stronger promoter was constructed, and this system provides a powerful tool for homologous or heterologous expression by using starch as a low-cost carbon source. Furthermore, our studies also lay a foundation for continued improvements in this expression system.

## Methods

### Strains and culture media

The strains listed in Table 1 were cultured on wheat bran extract slants at 30 °C to obtain fresh conidia. *Escherichia coli* DH5α was used for routine plasmid construction and amplification. Potato dextrose agar (PDA) medium and Vogel’s salts minimal medium (VMM) supplemented with 1% glucose or 1% (wt/vol) soluble starch were used for hyphal growth [15]. For phenotypic analyses of the parent strain and its mutants on agar plates, VMM supplemented with glucose as the sole carbon source was used, and 1 μl of conidia of each strain was spotted onto the medium and cultivated at 30 °C for 6 days.

**Table 1:** *P. oxalicum* strains used in this study

Strain name	Description	Parent strain	Reference
DB2	Δ*bgl2*-*bgl2*(p)::β-rec	M12	[32]
Δ13A	Δ*bgl2*-*bgl2*(p)::β-rec-Δ*amy13A*-*pyrG*	DB2	This study
Δ13A-OamyR	Δ*bgl2*-*bgl2*(p)::β-rec-Δ*amy13A*-*amy13A*(p)::*amyR*-*pyrG*	DB2	This study
Δ13A-OamyR-ΔCreA	Δ*bgl2*-*bgl2*(p)::β-rec-Δ*amy13A*-*amy13A*(p)::*amyR*-Δ*creA*-*pyrG*	Δ13A-OamyR	This study

To determine the amylase activity and biomass of different mutants, the strains were firstly grown in 100 mL of liquid GMM for 24 h. Then, their mycelia were collected by vacuum filtration, and 0.5 g of wet mycelia was resuspended in 100 mL of liquid VMM supplemented with 1% (wt/vol) starch. All plates were incubated in a 30 °C incubator, and all liquid cultures were grown in 300 mL flasks at 30 °C and 200 rpm. Mycelia were collected every 12 h and dried at 80 °C for 3 h to constant weight for biomass measurements.

### Targeting cassette construction and fungal transformation

The knockout or overexpression cassettes for each candidate gene were constructed by fusion PCR. The cassettes contained a 1.9-kb *pyrG* gene and DNA fragments approximately 2.0-kb upstream and downstream of the target gene, the DNA fragments were amplified by PCR using the corresponding primer pairs (Additional file 12: Table S11). Transformation of *P. oxalicum* was performed according to the method previously described [26]. The transformations identified in this study were further confirmed by PCR with specific primer pairs (Additional file 12: Table S11). The *pyrG* gene was excised by the β-rec/six self-excising marker recycling system, as reported previously [32].

### Enzyme assays, protein determination and SDS-PAGE analysis

Amylase activity was assayed according to the DNS method. The amounts of released reducing sugars were determined using the dinitrosalicylic acid method [26]. The absorbance of the reaction system was measured at 540 nm. Up to 1.5 mL of starch solution was added for amylase activity assays, and the reaction was incubated at 40 °C for 10 min. One unit of enzymatic activity (U) was defined as the amount of enzyme needed to release 1 μmol of glucose equivalent per minute. Protein concentration was measured by using a Bradford reagent kit (Sangon Biotech, China). SDS-PAGE was performed using 12% polyacrylamide to determine protein purity. The protein profile was analyzed by staining gels with Coomassie Brilliant Blue R-250 (Sangon Biotech, China) destaining gels with 10% (w/v) acetate solution.

### RNA isolation, cDNA synthesis and quantitative RT-PCR

For real-time PCR, after 8, 21, and 48 h of cultivation on starch, the mycelia were ground by using liquid nitrogen and total RNA was extracted using 1 mL of TRIzol reagent (TaKaRa, Japan) according to the manufacturer’s protocols. cDNA synthesis was performed using the PrimeScript RT Reagent Kit (Perfect Real Time) (TaKaRa, Japan) according to the manufacturer’s instructions. Quantitative PCR was performed using SYBR Premix Ex Taq^TM^ (Perfect Real Time) (TaKaRa, Japan). Three biological replicates and two experimental replicates were required for one sample. The actin gene was used as the internal standard. All primers are listed in Additional file 11: Table S11.

### RNA sequencing and transcription expression analysis

Fresh spores of each strain were inoculated into 100 mL of 1% glucose medium and incubated at 200 rpm and 30 °C for 24 h. Equal amounts of mycelia were transferred to 1% starch medium and cultured for 21 h. Total RNA was extracted from frozen *P. oxalicum* mycelia after lyophilization using the RNAiso^TM^ reagent (TaKaRa, Japan) and used to construct cDNA libraries. Transcriptome assays based on Illumina sequencing technology were performed at Shanghai Majorbio Bio-pharm Technology Co., Ltd (Shanghai, China). After quality control, the generated clean reads were mapped against predicted transcripts from the *P. oxalicum* 114-2 genome. Transcript abundance (fragments per kb per million reads, FPKM) genes with significantly different expression levels were identified through a significance test with combined thresholds (FDR ≤ 0.01 and fold change ≥ 2). Pearson’s correlation coefficient was used to evaluate transcriptome reliability and three biological replicates were used in each sample. Differential gene expression was analyzed by using the Majorbio cloud computing platform that included a series of DESeq software packages.

## Supplementary information

**Additional file** **1: Table S1**. Quality control statistics of transcriptomics sequences.

**Additional file 2: Table S2.** Gene list of the 680 genes showed increase in strain Δ13A-OamyR as compared to strain Δ13A.

**Additional file** **3: Table S3.** Gene list of the 1042 genes showed decrease in strain Δ13A-OamyR as compared to strain Δ13A.

**Additional file** **4: Table S4.** Gene list of the 762 genes showed increase in strain Δ13A-OamyR-ΔCreA as compared to strain Δ13A-OamyR.

**Additional file** **5: Table S5.** Gene list of the 935 genes showed decrease in strain Δ13A-OamyR-ΔCreA as compared to strain Δ13A-OamyR.

**Additional file** **6: Table S6.** Gene list of the 66 genes showed continuously increase in strains Δ13A, Δ13A-OamyR, Δ13A-OamyR-ΔCreA.

**Additional file** **7: Table S7.** Gene list of the 134 genes showed continuously decrease in strains Δ13A, Δ13A-OamyR, Δ13A-OamyR-ΔCreA

**Additional file** **8: Table S8.** List of 40 putative transcription factors genes determined in this study that response to overexpression of AmyR and deletion of CreA.

**Additional file** **9: Table S9**. *P. oxalicum* secretory components and their transcriptional responses to Amy15A overproduction.

**Additional file** **10: Table S10**. Transcriptional levels of some genes in the Δ13-OamyR-ΔCreA strain.

**Additional file** **11: Figure S1.** Expression levels of the *amy15A* genes in strains DB2, Δ13A, Δ13A-OamyR, Δ13A-OamyR-ΔCreA on glucose (-G) and starch (-S).

**Additional file** **12: Table S11.** Oligo nucleotide primers used for the study.

## Data Availability

All data generated or analysed during this study are included in this published article and its additional files.

## Abbreviations

DNS: Dinitrosalicyclic reactive oxygen species
SDS-PAGE: Sodium dodecyl sulfate–polyacrylamide gel electrophoresis
LC–MS/MS: Liquid chromatography-tandem mass spectrometry
TPM: Transcripts per million reads
CCR: Carbon catabolite repression
DEGs: Differentially expressed genes
