# Supplementary material for: Constructing a novel expression system by specific activation of amylase expression pathway in Penicillium
Source: Microb Cell Fact. 2020 Jul 29;19:155. doi: 10.1186/s12934-020-01410-4 (PMC7391575; doi:10.1186/s12934-020-01410-4)
Supplement: Supplementary file 10 — Additional file 10: Table S10. Transcriptional levels of some genes in the Δ13-OamyR-ΔCreA strain. [file 12934_2020_1410_MOESM10_ESM.docx]

Table S10 Transcriptional levels of some genes in the Δ13-OamyR-ΔCreA strain

| Gene ID | TPM | Description |
| --- | --- | --- |
| PDE_08540 | 37181.75 | Glucose-repressible protein |
| PDE_06605 | 26206.31 | Putative uncharacterized protein ART2 |
| PDE_06607 | 14909.86 | Hypothetical protein |
| PDE_07911 | 15933.57 | Hypothetical protein |
| PDE_09377 | 12434.23 | Sugar (and other) transporter |
| PDE_09417 | 12598.38 | Glucoamylase Amy15A |
| PDE_07106 | 11191.46 | Hypothetical protein |
| PDE_05655 | 11349.07 | Aegerolysin |
| PDE_07202 | 11230.98 | Glucose-repressible protein |
| PDE_02095 | 8139.33 | HSP20-like domain found in ArsA |
