# Supplementary figures and images for: Constructing a novel expression system by specific activation of amylase expression pathway in Penicillium
Source: Microb Cell Fact. 2020 Jul 29;19:155. doi: 10.1186/s12934-020-01410-4 (PMC7391575; doi:10.1186/s12934-020-01410-4)

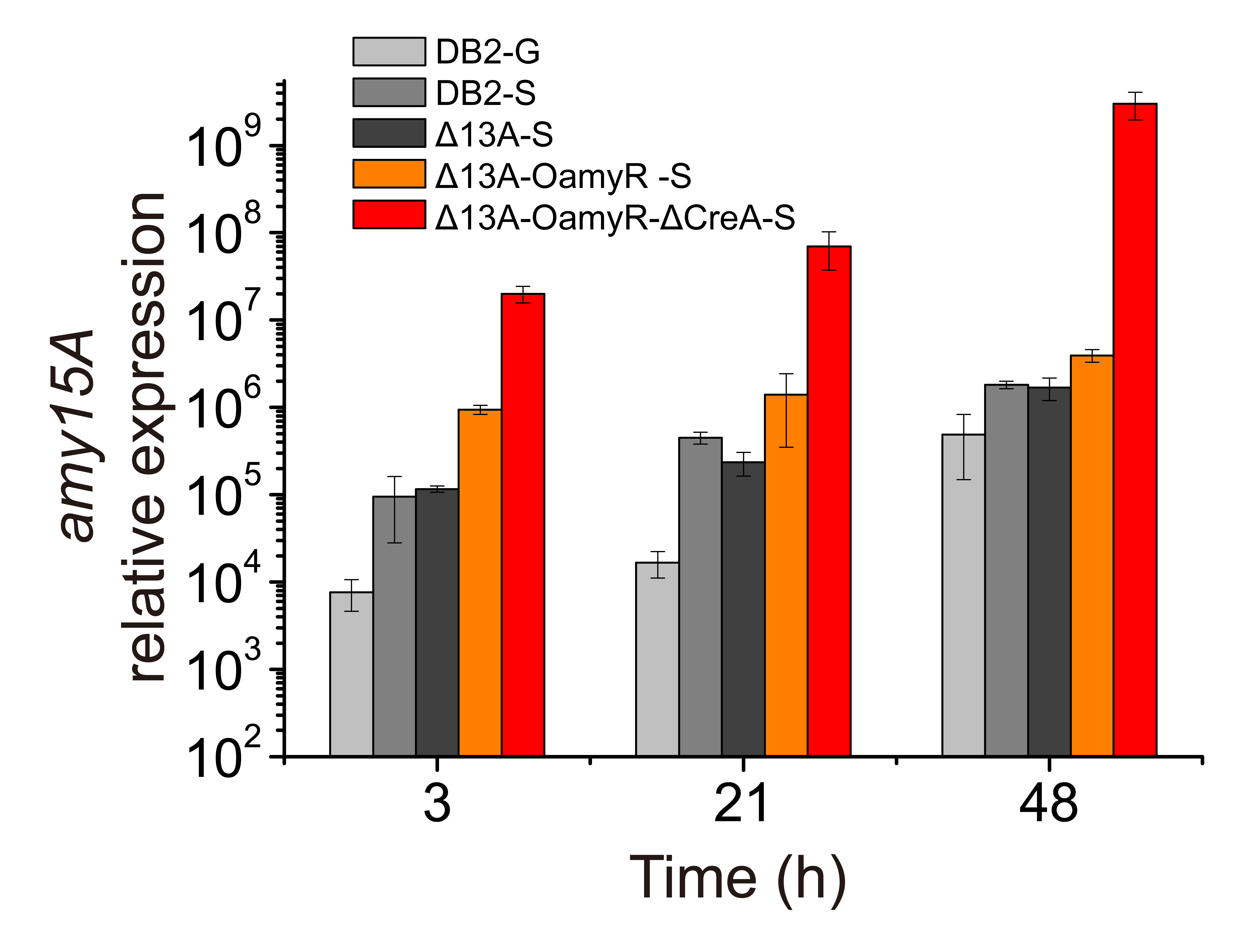

Supplement: Supplementary file 11 — Additional file 11: Figure S1. Expression levels of the amy15A genes in strains DB2, Δ13A, Δ13A-OamyR, Δ13A-OamyR-ΔCreA on glucose (-G) and starch (-S). [file 12934_2020_1410_MOESM11_ESM.tif]
