# Supplementary material for: Constructing a novel expression system by specific activation of amylase expression pathway in Penicillium
Source: Microb Cell Fact. 2020 Jul 29;19:155. doi: 10.1186/s12934-020-01410-4 (PMC7391575; doi:10.1186/s12934-020-01410-4)
Supplement: Supplementary file 12 — Additional file 12: Table S11. Oligo nucleotide primers used for the study. [file 12934_2020_1410_MOESM12_ESM.doc]

Primers used in this study.

| Primer name | Seqμence(5’-3’) |
| --- | --- |
| Amplification of *pyrG* coding sequence | |
| six-pyrG-F | GGAAGGATACAGTCGCTAGC |
| six-pyrG-R | CGTTCACACGTGAAGC |
| Construction of Δ13-OamyR | |
| P13A-up-F | AAGAGCGGACTGAGCATACG |
| amyR-pyrG-R | AGTTGCTAGCGACTGTATACCTTCCCAACTTGGGAGGCAGGAAAT |
| 13A-pyrG-F | TGCGGCCGCTTCACGTGTGAACGTATCAGGCAGAAGAGCACCGC |
| 13A-down-R | CTCGGAGTCGGGCGTTACAT |
| P13A-NF3 | CACATCACCCTGGAGTCTCGGATA |
| P13A-NR2 | GGTGGGCTGACGATTCTTGTAATT |
| Construction of Δ13 | |
| 13A×F | AAGAGCGGACTGAGCATACG |
| 13A-pyrG-R | AGTTGCTAGCGACTGTATACCTTCCATGCCTCCTGATGATACCACA |
| 13A-pyrG-F | TGCGGCCGCTTCACGTGTGAACGTATCAGGCAGAAGAGCACCGC |
| 13A-down-R | CTCGGAGTCGGGCGTTACAT |
| 13A×NF | GCCTTTTACCCCGCCATCGTTGCT |
| 13A×NR | GCCACAGGCGACAGTCATCGGTAA |
| Construction of Δ13-OamyR-ΔCreA | |
| crea-F | GGGCTCTTGAGGTTCATTGC |
| crea-pyrG-R | AGTTGCTAGCGACTGTATACCTTCCTTATCGGGATCGTGACAAGG |
| crea-pyrG-F | TGCGGCCGCTTCACGTGTGAACGCGTGGGCTCAGTGGTTCTA |
| crea-R | AAAGTGTCAGCAGGTCTCGG |
| crea-NF | ATACACCTAAACAAACACCCTCCGC |
| crea-NR | ATCCAGCAATTATATATGCAGGGCC |
| Verification of transformants | |
| pyrG-yz-F | TGCCCGAACAGAAAGAGG |
| Quantitative real-time PCR | |
| act-qF | CTCCATCCAGGCCGTTCTG |
| act-qR | CATGAGGTAGTCGGTCAAGTCAC |
| amy15A-qF | GGTCGGTTCTATTTCTCAGCTCG |
| amy15A-qR | ACTTGGCAGGGACGGTGTAGG |
| amyR-qF | ACCATCACCATCGGCAACT |
| amyR-qR | GAATGCGCTCGTGATGCTC |
| amy13A-qF | AGCATCAGCAGCCTTTACAA |
| amy13A-qR | GTTCAGACACGTAAGGCAAA |
